# Qualitative study of the association between psychosocial health and physical activity/sleep quality in toddlers

**DOI:** 10.1038/s41598-023-42172-4

**Published:** 2023-09-21

**Authors:** Chisa Tsuyuki, Koya Suzuki, Kanako Seo, Dandan Ke, Kyoko Tsuge, Pengyu Deng, Dajiang Lu, Hisashi Naito

**Affiliations:** 1 Graduate School of Health and Sports Science, Juntendo University, 1-1 Hirakagakuendai, Inzai, Chiba 270-1695 Japan; 2 Tokyo Research Laboratories, Kao Corporation, 2-1-3 Bunka, Sumida-Ku, Tokyo, 131-8501 Japan; 3 School of Public Health, Fudan University, Shanghai, 200032 China; 4 Tochigi Research Laboratories, Kao Corporation, 2606 Akabane, Ichikai-machi, Haga-gun, Tochigi 321-3497 Japan; 5 School of Exercise and Health, Shanghai University of Sport, Shanghai, 200438 China

**Keywords:** Health care, Paediatrics

## Abstract

Physical activity and sleep are important factors of mental and physical health in children, with some reports indicating that their effects can persist into adulthood. However, there is limited research on the qualitative aspects of physical activity and sleep in preschool children, particularly in those aged < 3 years. Therefore, to elucidate the association between psychosocial health and physical activity/sleep in early childhood in terms of qualitative aspects, we conducted a retrospective cohort study in 2985 3-year-old children (37.2 ± 0.75 months) in Shanghai, China. An analysis using structural equation modeling indicated that current physical activity had a direct and moderate impact on current psychosocial health evaluated using the Strength and Difficulties Questionnaire. In particular, past physical activity had an indirect and mild effect on current psychosocial health via current physical activity in girls. However, regardless of sex, past sleep quality had slight impact on current psychosocial health, not only indirectly via current sleep quality, but also directly. These findings highlight the importance of considering the qualitative aspects of physical activity and sleep quality as significant factors influencing the current and future psychosocial health of children, even at a very early age (< 3 years).

## Introduction

Physical activity and sleep are crucial for both physical and mental health in infants and toddlers. The World Health Organization (WHO) introduced the “24-Hour Movement Guidelines for the Early Years” in 2019^1^, which state “for the greatest health benefits, infants, and young children should meet all the recommendations for physical activity, sedentary behavior, and sleep in a 24-h period.” The guidelines vary based on age groups; for example, for 1–2-year-olds, the recommendations include a minimum of 180 min of physical activity, 11–14 h of sleep, and maximum sedentary screen time of up to 60 min for 2-year-olds and 0 min for 1-year-olds. For 3–4-year-olds, the guidelines recommend at least 180 min of physical activity, including 60 min of moderate-to-strenuous activity, 10–13 h of sleep, and sedentary screen time of up to 60 min or less.

The guidelines were developed in response to the social issues observed in children in recent years, such as the increase in obesity rates due to increased sedentary behaviors and lack of physical activity^2–4^, lack of physical activity due to obesity^5^ and increased screen time in children^6^, deterioration of sleep quality due to increased screen time^7^, and concerns about the impact of these lifestyle changes on mental and physical health. According to a flagship report by the United Nations Children's Fund^8^, the global proportion of overweight (obese) children under the age of 5 years increased from 4.8% in 1990 to 5.9% in 2018 due to increased calorie intake and dietary changes as well as changes in lifestyle and behaviors due to rapid economic development, urbanization, and technological advancements. Cheung et al*.*^9^ found a significant association between the frequency of touchscreen use and sleep volume (reduced total duration, reduced night-time sleep, and increased daytime sleep) and delayed sleep onset (time taken to fall asleep) based on data from 715 infants aged 6–36 months in the United Kingdom. Recently, the negative impact of restrictions on physical and social activity due to the spread of coronavirus disease 2019 (COVID-19) and the decrease in opportunities for human contact on children's lifestyles has become a concern^10^. A study conducted in Shanghai, China^11^, revealed that children who were confined to their homes for approximately 2 months due to COVID-19-related school closures experienced sleep disorders, lack of exercise, excessive media viewing, lack of parental care, mental health problems of parents, and harsh parenting, which greatly affected the children's mental health.

Numerous previous studies have highlighted the significance of basic lifestyle components, such as physical activity and sleep, during childhood and their association with physical and mental health. A previous study^12^ of 3–6-year-olds in China used the Strength and Difficulties Questionnaire (SDQ)/Clancy Autism Behavior Scale, screen time, and night-time sleep duration and showed that toddlers with prolonged screen time and shorter sleep duration (less than the intra-sample average of 9.15 h) were significantly more likely to have behavioral problems. Furthermore, previous research has revealed that toddlers’ current lifestyle components, such as physical activity and sleep, are influenced by their past lifestyle. Regarding the amount of physical activity and strength in childhood, Boreham and Ridoch^13^ noted that the benefits of physical activity in childhood include a biological carryover effect in adulthood (improved health in adulthood) and that such behaviors are carried over to adulthood (active children are more likely to be active and healthy in adulthood than children who are inactive), suggesting that physical activity in childhood affects physical activity and strength in adulthood. Chen et al.^6^ conducted a study on behavior of children in Singapore using screen time data in children aged 2–3 years and wrist-worn accelerometer in children aged 5.5 years for 3–7 days; the findings revealed that toddlers who had a total screen time of more than 3 h at the age of 2–3 years had increased sedentary behavior at the age of 5.5 years and less physical activity at both mild and medium levels compared with that noted toddlers who had a total screen time of 1 h or less. Therefore, it is evident that children's basic lifestyle components, particularly physical activity and sleep, not only influence their current well-being but also have implications for their future lifestyle, physical, and mental health, possibly extending into adulthood. However, only few studies^14–16^ have clarified the impact of these effects on the physical/mental health of preschool-age children, and the extent to which lifestyle components, especially in children younger than 3 years, affect their current and future mental/physical health remains unclear. Furthermore, just as the 24-Hour Movement Guidelines for the Early Years developed by the WHO are limited to quantitative recommendations on the time devoted to maintain a specific lifestyle, these lifestyles components are often evaluated from quantitative aspects. Regarding sleep^17^, some reports suggest that quality is a better evaluation index than quantity to support the healthy growth of children. Therefore, it is essential to explore these effects in childhood not only from a quantitative but also a qualitative perspective.

Therefore, this study aimed to clarify the effects of lifestyle components on psychosocial health in early childhood from qualitative aspects other than quantity. We conducted a survey on 3022 3-year-old children living in Shanghai, China, about their past (at the age of 1 year) and current (at the age of 3 years) lifestyle and psychosocial health. Particularly, in accordance with the 24-Hour Movement Guidelines developed by the WHO, among many lifestyle components, we focused on physical activity, including motor skills (both gross and fine motor movements), and sleep quality. We formulated and verified two hypotheses: (1) physical activity at the ages of 1 and 3 years affects psychosocial health at the age of 3 years, and (2) sleep quality at the ages of 1 and 3 years affects psychosocial health at the age of 3 years.

## Results

### Analyzing causality by structural equation modeling

**Figure 1:**
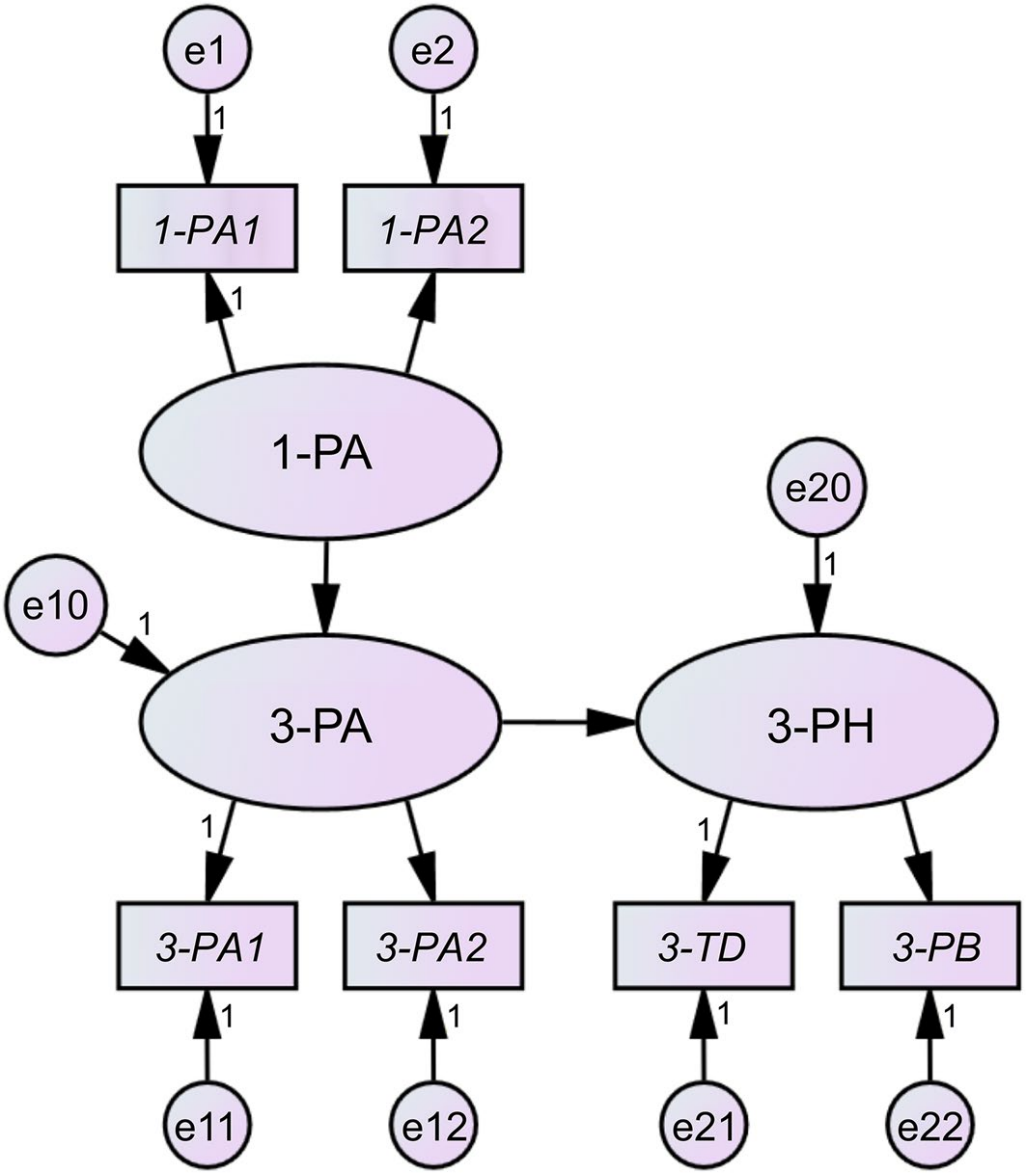
Pathways linking physical activity and psychosocial health. PA: physical activity, PH: psychosocial health, TD: total difficulties, PB: prosocial behavior; e: error term (especially, e10 and e20 in this model mean disturbance terms). 1-: at the age of 1 year; 3-: at the age of 3 years. PA1: often moved around freely by themselves; PA2: often played with their hands.

Figure 1 displays the final model adopted to illustrate the relationship between physical activity and psychosocial health. Table 1 presents the fit indices, path coefficients, and significance probabilities for each model. The fit indices of the mixed-sex model with 2985 toddlers were as follows: goodness-of-fit index (GFI) = 0.999, adjusted goodness-of-fit index (AGFI) = 0.997, comparative fit index (CFI) = 0.991, root mean square error of approximation (RMSEA) = 0.011, and Akaike information criterion (AIC) = 37.569. All the path coefficients were statistically significant (*p* < 0.05). This model’s fit indices showed significantly superior values across all criteria compared with that noted for other models; therefore, we judged it as the model that best explains the causal structure of physical activity and psychosocial health at the age of 1 and 3 years. The path coefficient from physical activity at the age of 1 year to physical activity at the age of 3 years was 0.535, and that from physical activity at the age of 3 years to psychosocial health at the age of 3 years was 0.579. The fit indices of the model for the 1476 boys were as follows: GFI = 0.997, AGFI = 0.990, CFI = 0.908, RMSEA = 0.029, and AIC = 43.424. The path coefficient from physical activity at the age of 1 year to 1-P2 and from physical activity at the age of 1 year to physical activity at the age of 3 years did not show statistically significant values. The path coefficient from physical activity at the age of 3 years to psychosocial health at the age of 3 years was 0.517, which was statistically significant (*p* < 0.05). The fit indices of the model for the 1509 girls were as follows: GFI = 1.000, AGFI = 0.999, CFI = 1.000, RMSEA = 0.000, and AIC = 30.022. The path coefficient from physical activity at the age of 1 year to physical activity at the age of 3 years was 0.666, and the path coefficient from physical activity at the age of 3 years to psychosocial health at the age of 3 years was 0.594. All path coefficients showed statistically significant values (*p* < 0.05).

**Table 1:** Goodness-of-fit indices and path coefficients of each structural equation model for physical activity and psychosocial health.

	Total			Boys			Girls		
GFI	0.999			0.997			1.000		
AGFI	0.997			0.990			0.999		
CFI	0.991			0.908			1.000		
RMSEA	0.011			0.029			0.000		
AIC	37.569			43.424			30.022		
	*Β*	*β*	Sig	*Β*	*β*	Sig	*Β*	*β*	Sig
1-PA → 3-PA	0.585	0.535	***	0.296	0.330	0.070	0.805	0.666	***
3-PA → 3-PH	3.338	0.579	***	2.892	0.517	*	3.333	0.594	***
1-PA → 1-PA1	1.000	0.389	–	1.000	0.387	–	1.000	0.407	–
1-PA → 1-PA2	0.965	0.349	***	0.910	0.334	0.096	0.939	0.350	***
3-PA → 3-PA1	1.000	0.390	–	1.000	0.321	–	1.000	0.450	–
3-PA → 3-PA2	0.896	0.324	***	1.142	0.335	**	0.802	0.338	***
3-PH → 3-TD	1.000	0.376	–	1.000	0.293	–	1.000	0.433	–
3-PH → 3-PB	0.436	0.398	***	0.565	0.416	*	0.405	0.410	***

Path coefficients: partial regression coefficient (*B*), standardized partial regression coefficient (*β*).

Parameter estimation: maximum likelihood.

***p < 0.001; **p < 0.01; *p < 0.05.

GFI: goodness-of-fit index, AGFI: adjusted goodness-of-fit index, CFI: comparative fit index, RMSEA: root mean square error of approximation, AIC: Akaike information criterion, PA: physical activity, PH: psychosocial health, TD: total difficulties, PB: prosocial behavior.

1-: at the age of 1 year, 3-: at the age of 3 years, PA1: often moved around freely by themselves, PA2: often played with their hands.

**Figure 2:**
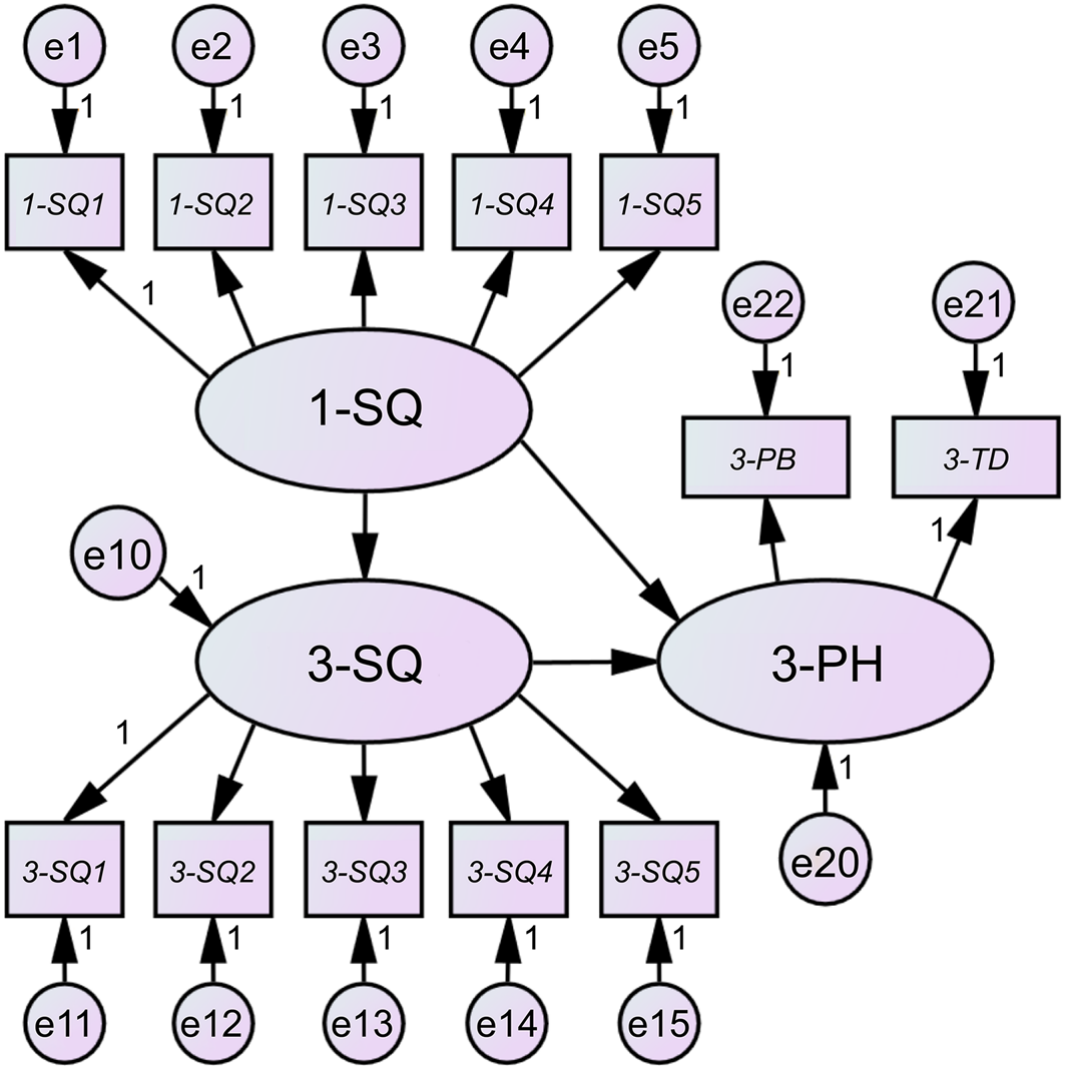
Pathways linking sleep quality and psychosocial health. SQ: sleep quality, PH: psychosocial health, TD: total difficulties, PB: prosocial behavior; e: error term (especially, e10 and e20 in this model mean disturbance terms). 1-: at the age of 1 year; 3-: at the age of 3 years. SQ1: experienced difficulty in putting them to sleep; SQ2: cried at midnight; SQ3: had irregularities in sleeping and wake-up times; SQ4: woke up often in the middle of the night; SQ5: often had a bad mood during the day.

Figure 2 shows the final model ultimately adopted for examining the relationship between sleep quality and psychosocial health. Table 2 shows the fit indices, path coefficients, and significance probabilities for each model. The fit indices of the mixed-sex model of 2985 toddlers were as follows: GFI = 0.988, AGFI = 0.982, CFI = 0.967, RMSEA = 0.033, and AIC = 268.580. This model's fit indices had significantly superior values across all criteria, and we judged it as the model that best explains the causal structure of sleep quality at the age of 1 and 3 years and psychosocial health at the age of 3 years. The path coefficient from quality of sleep quality at the age of 1 year to sleep quality at the age of 3 years was 0.304, that from sleep quality at the age of 1 year to psychosocial health at the age of 3 years was 0.142, and that from sleep quality at the age of 3 years to psychosocial health at the age of 3 years was 0.413. The fit indices of the model for the 1476 boys were as follows: GFI = 0.985, AGFI = 0.978, CFI = 0.965, RMSEA = 0.033, and AIC = 186.809. The path coefficient from sleep quality at the age of 1 year to sleep quality at the age of 3 years was 0.304, that from sleep quality at the age of 1 year to psychosocial health at the age of 3 years was 0.137, and that from sleep quality at the age of 3 years to psychosocial health at the age of 3 years was 0.426. The fit indices of the model for 1509 girls were as follows: GFI = 0.987, AGFI = 0.980, CFI = 0.973, RMSEA = 0.030, and AIC = 173.407. The path coefficient from sleep quality at the age of 1 year to sleep quality at the age of 3 years was 0.301, that from sleep quality at the age of 1 year to psychosocial health at the age of 3 years was 0.148, and that from sleep quality at the age of 3 years to psychosocial health at the age of 3 years was 0.401. In all three models, the path coefficients had statistically significant values (*p* < 0.05).

**Table 2:** Goodness-of-fit indices and path coefficients of each structural equation model for sleep quality and psychosocial health.

	Total			Boys			Girls		
GFI	0.988			0.985			0.987		
AGFI	0.982			0.978			0.980		
CFI	0.967			0.965			0.973		
RMSEA	0.033			0.033			0.030		
AIC	268.580			186.809			173.407		
	*Β*	*β*	Sig	*Β*	*β*	Sig	*Β*	*β*	Sig
1-SQ → 3-SQ	0.242	0.304	***	0.255	0.304	***	0.228	0.301	***
3-SQ → 3-PH	2.401	0.413	***	2.277	0.426	***	2.512	0.401	***
1-SQ → 3-PH	0.658	0.142	***	0.614	0.137	*	0.704	0.148	***
1-SQ → 1-SQ1	1.000	0.638	–	1.000	0.610	–	1.000	0.666	–
1-SQ → 1-SQ2	1.120	0.688	***	1.191	0.693	***	1.059	0.685	***
1-SQ → 1-SQ3	0.871	0.562	***	0.930	0.573	***	0.819	0.552	***
1-SQ → 1-SQ4	1.013	0.660	***	1.065	0.664	***	0.966	0.655	***
1-SQ → 1-SQ5	0.777	0.540	***	0.849	0.561	***	0.715	0.520	***
3-SQ → 3-SQ1	1.000	0.530	–	1.000	0.520	–	1.000	0.537	–
3-SQ → 3-SQ2	1.018	0.568	***	1.004	0.551	***	1.041	0.586	***
3-SQ → 3-SQ3	0.936	0.490	***	0.953	0.491	***	0.924	0.489	***
3-SQ → 3-SQ4	0.912	0.502	***	0.896	0.482	***	0.935	0.524	***
3-SQ → 3-SQ5	0.607	0.360	***	0.585	0.350	***	0.634	0.371	***
3-PH → 3-TD	1.000	0.655	–	1.000	0.583	–	1.000	0.724	–
3-PH → 3-PB	0.144	0.229	***	0.142	0.209	**	0.145	0.245	***

Path coefficients: partial regression coefficient (*B*), standardized partial regression coefficient (*β*).

Parameter estimation: maximum likelihood.

*** *p* < 0.001, **:*p* < 0.01, * *p* < 0.05.

GFI: goodness-of-fit index, AGFI: adjusted goodness-of-fit index, CFI: comparative fit index, RMSEA: root mean square error of approximation, AIC: Akaike information criterion, SQ: sleep quality, PH: psychosocial health, TD: total difficulties, PB: prosocial behavior.

1-: at the age of 1 year; 3-: at the age of 3 years.

SQ1: experienced difficulty in putting them to sleep; SQ2: cried at midnight; SQ3: had irregularities in sleeping and wake-up times; SQ4: woke up often in the middle of the night; SQ5: often had a bad mood during the day.

## Discussion

In this study, we tested two hypotheses using structural equation modeling: (1) physical activity at the ages of 1 and 3 years affects psychosocial health at the age of 3 years and (2) sleep quality at the ages of 1 and 3 years affects psychosocial health at the age of 3 years. In the model of physical activity and psychosocial health, the path coefficient from physical activity at the age of 1 year to physical activities at the age of 3 years was 0.535 and that from physical activity at the age of 3 years to psychosocial health at the age of 3 years was 0.579, both of which were statistically significant. This result indicates that, although no direct effect of physical activity at the age of 1 year on psychosocial health at the age of 3 years was confirmed, the indirect effect from physical activity at the age of 1 year on psychosocial health at the age of 3 years via physical activity at the age of 3 years was 0.535 × 0.579 = 0.309. In particular, the association was more pronounced when only girls were analyzed; the path coefficient from physical activity at the age of 1 year to physical activity at the age of 3 years was not significant when only boys were analyzed. However, the model for sleep quality and psychosocial health showed path coefficients of 0.304 from sleep quality at the age of 1 year to sleep quality at the age of 3 years, 0.141 from sleep quality at the age of 1 year to psychosocial health at the age of 3 years, and 0.410 from sleep quality at the age of 3 years to psychosocial health at the age of 3 years. All the paths were statistically significant. That is, the direct effect from sleep quality at the age of 1 year to psychosocial health at the age of 3 years was 0.141, and the indirect effect from sleep quality at the age of 1 year to psychosocial health at the age of 3 years via sleep quality at the age of 3 years was 0.304 × 0.410 = 0.124; the combined direct and indirect effects were 0.265. When analyzed by sex, no significant difference was noted in the results.

Regarding the causal relationship between physical activity and psychosocial health, in the mixed-sex model and model that analyzed only girls, the associations both for “from physical activity at the age of 1 year to physical activity at the age of 3 years” and “from physical activity at the age of 3 years to psychosocial health at the age of 3 years” showed moderate strength. In contrast, in the model that included only boys, the association for “from physical activity at the age of 3 years to psychosocial health at the age of 3 years” showed moderate strength, but that for "from physical activity at age 1 year to physical activity at age 3 years" was not significant. These results indicate that physical activity at the age of 3 years had direct and moderate effect on psychosocial health at the age of 3 years regardless of sex. Particularly in girls, since physical activity at the age of 1 year had a moderate effect on physical activity at the age of 3 years, physical activity at the age of 1 year showed an indirect and mild effect on psychosocial health at the age of 3 years via physical activity at the age of 3 years. In addition, the two factors “often moved around freely by themselves" and "often played with their hands,” defined as “physical activity” in this study, showed equal importance for the psychosocial health of toddlers, the values of the path coefficient from “physical activity” were approximately the same. These results under the analytical conditions of this study indicated that the hypothesis that “physical activity at the ages of 1 and 3 years affects psychosocial health at the age of 3 years” held true when boys and girls were combined or only girls were analyzed, while it did not partially hold true when only boys were analyzed.

Previous studies^12, 13^ have suggested that quantitative factors such as physical activity time affect current and future physical/mental health; however, the present study suggests the importance of not only quantitative but also qualitative elements as crucial factors affecting psychosocial health. In this study, physical activity was defined based on two items: gross motor movement involving the entire body and fine motor movement using fingertips and hands. Other studies^18, 19^ have highlighted the impact of childhood experiences, such as the frequency of outdoor play and engaging in risky play (adventurous play), on not only later physical activity but also social skills in adolescence. Fischer et al.^20^ evaluated the fine physical activity of preschoolers in three tasks: placing a coin into a box hole, passing a string through a bead, and tracing a line with a pen. Their findings suggested associations between these fine motor skills and numerical skills and mathematical development. However, previous studies have stated the importance of encouraging children to perform voluntary and active actions. Hewes^21^ states that spontaneous free play contributes to children's physical health (strength, cooperativeness, spatial awareness, balance) and flexibility as well as resilience and adaptability in emotional and social responses. Based on the above information, in order to support the healthy physical and mental growth of children, it might be important to not only recommend time guidelines and relatively large physical activity but also convey the importance of performing a variety of exercises and play activity from an early age regardless of gross or fine motor movement and to enhance an environment in which children can voluntarily and actively perform these activities.

Next, we discuss the causal structures between sleep quality and psychosocial health. Both the mixed-sex and sex-specific models showed weak associations for “from sleep quality at the age of 1 year to sleep quality at the age of 3 years” and moderate association for “from sleep quality at the age of 3 years to psychosocial health at the age of 3 years.” Furthermore, the path of “from sleep quality at the age of 1 year to psychosocial health at the age of 3 years” showed a significant association although not that large, with a total direct and indirect effect of 0.266. Thus, regardless of sex, the results suggest that sleep quality at the age of 3 years may have a direct and moderate effect on psychosocial health at the age of 3 years, and sleep quality at the age of 1 year may have slight impact on psychosocial health at the age of 3 years, not only indirectly via sleep quality at the age of 3 year, but also directly. In particular, among the explanatory variables of psychosocial health (total difficulties and prosocial behavior) the path coefficient for total difficulties was significantly higher than that for prosocial behavior. These results suggest that sleep quality not only at the age of 3 years but also at a very young age of 1 year, is a factor that affects psychosocial health, especially total difficulties, both in the present and in the future. These results under the analytical conditions of this study mean that the hypothesis “sleep quality at the ages of 1 and 3 years affects psychosocial health at the age of 3 years” was true regardless of sex.

Numerous studies have focused on sleep quality in adults. Some studies^22, 23^ reported that the quality of adult sleep is affected by daytime stress, screen time before falling asleep, and sleep comfort. Although few studies have reported on sleep in healthy children, the guidelines of the National Sleep Charity in the United Kingdom^24^ and the Centers for Disease Control and Prevention in the United States^25^ state that a comfortable environment is as important for better sleep for children as adults. These guidelines include, for example, a quiet and comfortable bedroom, active movement during the day, and removal of electronic devices such as TVs and smartphones from the bedroom. Since this study suggested that sleep quality at a very early stage, about the age of 1 year, affects future sleep quality and even psychosocial health, improving sleep quality about the age of 1 year may be important for improving sleep quality in the future and maintaining psychosocial health. Therefore, to support the healthy physical and mental growth of children regarding sleep quality, similar to physical activity, it might be necessary to provide not only time guidelines (10–14 h per day is recommended for 1–4-year-olds) but also specific examples that caregivers can follow to improve sleep quality, such as sleepwear and room temperature settings. Some previous studies^26–28^ have reported that newborns particularly have no circadian rhythm or a fixed sleep cycle, that the circadian rhythm and nocturnal sleep patterns develop at approximately 10–12 weeks, and that the sleep–wake patterns are driven by complex interactions between physiological processes and environmental, behavioral, and social factors, and that individual differences are there too. Hence, when providing specific examples to caregivers of practicable to improve the quality of children's sleep, accurate explanations are necessary to ensure that they do not worry excessively about children crying/waking at night.

Previous studies have also reported an association between physical activity and sleep. Gibson et al.^29^ reported that sleep timing and efficiency are significantly associated with cognitive and fine motor development in children aged 11–14 months. Several previous studies^30, 31^ have also reported an association between moderate amounts and intensity of physical activity during the day and rhythms of life, including sleep. Therefore, the physical activity and sleep quality analyzed in this study may be correlated. In this study, in order to explore the association between lifestyle and psychosocial health from aspects other than quantity, we focused on physical activity and sleep in accordance with the WHO 24-Hour Movement Guidelines for the Early Years from among many lifestyle components. Diet is often cited as an important factor for the healthy growth of children^32–35^, in addition to physical activity and sleep. A report^36^ on the adherence to the Vegetable and Fruit Intake Guidelines issued by the Australian Department of Health and the National Committee for Health and Medical Researchers stated that diet is significantly associated with low overall SDQ difficulty scores. These studies suggest that diet, as well as physical activity and sleep, is one of the key lifestyle components that can affect psychosocial health. Therefore, it might be valuable to investigate the association between physical activity and sleep and the association between childhood diet and psychosocial health in future studies.

Some reports state that children's lifestyles are greatly influenced by the lifestyle and child-rearing environment around them. Fertig et al.^37^ showed that children with full-time employed mothers tend to sleep for approximately 50 min lesser, on average, per weekend day than children with stay-at-home mothers did. Dubois-Comtois et al.^38^ suggested that children's sleep time is related to maternal factors, given that maternal anxiety is directly transmitted to children, and instable relationships with mothers are likely to lead to insomnia in children. In addition, a study conducted by Wang involving 5–6-year-old kindergarteners in China^39^ reported that provision of additional physical education classes over a 3-month period improved their communication skills and critical thinking scores. In other words, in order to support basic lifestyles such as physical activity and sleep in childhood, it might be important for society as a whole to promote support that focus on the children as well as their surrounding environment, such as their caregivers and childcare facilities.

This study has some limitations. The first limitation is the sampling bias, given that the participants were invited to participate. As this survey included mothers and children living in Shanghai, China, further research is required to determine whether similar trends can be observed in other parts of China and in other countries. In addition, we accepted mothers/toddlers who could visit the designated examination venue on a predetermined date and excluded those who had respiratory symptoms or lived with someone who had symptoms in the previous 2 weeks. These inclusion/exclusion criteria may also have had a sampling bias. The second limitation is the analytical limitations of structural equation modeling. In this study, two question items assuming gross motor movement and fine motor movement were defined as explanatory variables of physical activity, and five question items based on the Japanese Sleep Questionnaire for Preschoolers (JSQP) were defined as explanatory variables for sleep quality. If the questions defined as explanatory variables for each factor and their content change, the association shown in this study may change. Third, all responses in the study were provided by the participants’ mothers, who were asked to report on their children's lifestyle and psychosocial health status. This reliance on maternal reports may introduce a certain degree of subjectivity and may not provide an entirely objective evaluation of the toddlers based on uniform criteria. In addition, since this was a retrospective cohort study, some questions about the past, such as the events at birth or at 1 year of age, were answered based on the mothers’ memories. Although we confirmed that the self-made questionnaires were moderately reproducible, the responses obtained in this manner may still be influenced by maternal subjectivity (about their children and parenting) and recall ambiguity. Consequently, it is essential to further explore how the qualitative data gathered in this study relates to objectively and quantitatively evaluated indicators in future research.

In conclusion, this retrospective cohort study of 3022 3-year-old children, including 2985 valid participants, living in Shanghai, China, and their mothers, aimed to qualitatively analyze the association between childhood lifestyle and psychosocial health. In particular, the study focused on physical activity assuming motor skills (both gross and fine motor movement) and sleep quality among many lifestyle components; we hypothesized that (1) physical activity at the ages of 1 and 3 years affects psychosocial health at the age of 3 years, and (2) sleep quality at the ages of 1 and 3 years affects psychosocial health at the age of 3 years. We verified those two hypotheses using structural equation modeling. As a result, the hypothesis (1) regarding physical activity held true when considering both boys and girls together, and also when analyzing only girls; however, it did not hold to be completely true when analyzing only boys. In contrast, the hypothesis (2) on sleep quality held true regardless of sex. These results indicate that physical activity and sleep in early childhood could be important factors that lead to psychosocial health, not only for quantitative but also in qualitative elements, and that they are some of the factors that affect the current and future conditions from a very early age, such as before the age of 3 years. This study suggests that, in addition to the generally indicated temporal guidelines, it is beneficial to support children's physical and mental health in aspects other than quantity, such as diversity of exercise and play and improvement in sleep quality.

## Methods

### Participants

A total of 3022 (1498 boys and 1524 girls) 3-year-old (36–38 months) children residing in Shanghai, China, participated in this survey with their mothers (aged 25–33 years). This study was a retrospective cohort study, and some questions about the events at birth and 1 year of age were answered by the mothers while referring to the Maternal and Child Health Handbook records, childcare record applications, and recalling memories 2–3 years ago. All questionnaires were in Chinese, and the time required to complete all questionnaires was approximately 60 min. To reduce the incidence of unfilled answers as much as possible and to collect accurate data, the questionnaire was completed in person at a designated venue, and the participants were accompanied by a staff measurer. Nevertheless, participants whose answers were less credible were excluded. Regarding credibility of the responses, if all the responses to two or more main questions consisting of 10 or more sub-questions were identical, the response to the main question was judged to be of low credibility, and the response to the relevant part was invalidated. Therefore, the number of valid respondents finally included in this study was 2985 (1476 boys and 1509 girls). Table 3 shows the basic information of the valid respondents. The data for height and weight were obtained from the mothers' records, as direct measurements were not taken at the survey venue. The height and weight at birth and at the ages of 1 and 3 years were similar to those reported in previous studies^40, 41^.

**Table 3:** Study participant characteristics stratified by sex.

Characteristic	Total (N = 2985) *Mean* ± *SD*	Boys (N = 1476) *Mean* ± *SD*	Girls (N = 1509) *Mean* ± *SD*	Sig	d
Age (months)	37.20 ± 0.75	37.24 ± 0.74	37.17 ± 0.76	*	0.080
Birth height (cm)	49.66 ± 1.18	49.84 ± 1.18	49.48 ± 1.16	**	0.332
Birth weight (kg)	3.10 ± 0.29	3.12 ± 0.29	3.08 ± 0.28	**	0.082
1—height (cm)	75.29 ± 2.64	75.67 ± 2.53	74.92 ± 2.69	**	0.463
1—weight (kg)	10.10 ± 0.57	10.21 ± 0.54	10.00 ± 0.58	**	0.280
3—height (cm)	94.78 ± 2.94	95.06 ± 2.90	94.50 ± 2.96	**	0.324
3—weight (kg)	14.93 ± 1.53	15.01 ± 1.49	14.85 ± 1.57	**	0.127

Significant differences (*p < 0.05, **p < 0.01) between the sexes.

SD: standard deviation, sig.: significance, d: Cohen’s d (effect size).

1-: at the age of 1 year; 3-: at the age of 3 years.

The survey was conducted from December 2020 to September 2021. The selection criteria for eligibility were as follows: (1) toddlers aged from 36 to 38 months; (2) mothers and toddlers residing in Shanghai since the birth of the target toddler to the time of the survey; (3) mothers and toddlers who could visit the designated examination venue on a predetermined date; and (4) mothers who agreed to answer questions, such as height, weight, educational background, annual income, medical history, disability, size of parental residence and medical history of the target toddler, to confirm that the population was not singular. The exclusion criteria were as follows: (1) toddler or mother having fever or respiratory symptoms during the 2 weeks prior to the visit to the test center and (2) a family member living with the toddler or mother, having fever or respiratory symptoms during the 2 weeks prior to the visit to the test center. The recruitment methods were solicitation at pediatric hospitals and kindergartens (approximately 20%), online shop sites or stores (approximately 10%), friend circles for mothers on social networking sites (approximately 20%), and referrals from friends of mothers (approximately 50%).

### Ethics declarations

This study was conducted in accordance with the guidelines proposed in the Declaration of Helsinki, and the research protocol was approved by the Human Research Ethics Committee of Kao Corporation (research number T206-190220), Ethics Committee of Juntendo University (No. 2020-16) and Shanghai Nutrition Society Ethics Committee ([2020] No. 012) (UMIN000042111). Written informed consent was provided by the parents of the participants.

### Data collection and analysis

All survey questionnaires were distributed as hard copies, and mothers responded by referring to the maternal and child health handbook and childcare record applications. Psychosocial health was assessed using the SDQ, a standardized scale that assesses psychosocial health from two perspectives: strength and difficulties. The SDQ comprises five subscales, with four of them assessing difficulties: emotional symptoms, conduct problems, hyperactivity/inattention, and peer relationship problems; these four subscales together form the total difficulties. The remaining subscale evaluates strength: prosocial behavior. Each of the five subscales consists of five items, making a total of 25 question items in the SDQ. Participants responded to each item using one of three answer choices: “not true” (0 point), “somewhat true” (1 point), and “certainly true” (2 points). The total score for each subscale is 0–10 points. Therefore, for prosocial behavior, scores range from 0 to 10 points, while total difficulties, which include the four difficulty subscales, have scores ranging from 0 to 40 points. A higher score on the prosocial behavior subscale (for strength) indicates better strengths and conditions, whereas lower scores on the difficulty subscales and total difficulties (for difficulties) indicate lower levels of difficulty and better overall psychosocial well-being/condition. Table 4 presents the scores for each SDQ category. No significant differences were noted in the responses between boys and girls for each SDQ category. In addition, when comparing these scores with the scores of previous studies^12, 42^ that evaluated the targets, including 3-year-olds, using the SDQ, no specificity (specific patterns or trends) in scores nor the tendency of responses was noted for each category.

**Table 4:** Mean subscale scores by sex for the parent-completed SDQ.

SDQ Scale Parent	Total (N = 2985) *Mean* ± *SD*	Boys (N = 1476) *Mean* ± *SD*	Girls (N = 1509) *Mean* ± *SD*	Sig	d
Emotional symptoms	3.02 ± 1.76	3.01 ± 1.79	3.02 ± 1.74	ns	0.009
Conduct problems	3.07 ± 1.54	3.10 ± 1.55	3.03 ± 1.53	ns	0.055
Hyperactivity—inattention	4.70 ± 1.68	4.72 ± 1.68	4.68 ± 1.69	ns	0.028
Peer problems	3.31 ± 1.59	3.34 ± 1.60	3.28 ± 1.58	ns	0.049
Prosocial behavior	5.74 ± 1.69	5.72 ± 1.66	5.75 ± 1.71	ns	0.024
Total difficulties	14.10 ± 4.09	14.17 ± 4.18	14.02 ± 4.00	ns	0.077

Significant differences between sex groups.

SDQ, Strength and Difficulties Questionnaire, SD: standard deviation; sig.: significance; ns: no significance; d: Cohen’s d (effect size).

Regarding physical activity and sleep quality, qualitative questions were asked on a 4-point scale for both the ages of 1 (past) and 3 (current) years. For physical activity (both gross and fine motor movement), two questions were set to evaluate the balance of physical activity: PA1: whether your child often move around freely by themselves, PA2: whether your child often plays with their hands. Sleep quality was assessed referring the JSQP^43^, which is mainly used to detect sleep disorders at an early stage in preschoolers^44^. The JSQP comprises 39 questions; however, certain questions were not applicable to the specific purpose of this study, such as those focusing on detection of diseases and those involving a quantitative viewpoint. As a result, these irrelevant questions were excluded, and five questions were defined from a general perspective that could be easily answered for both 1- and 3-year-olds. The contents of these questions are provided in the legend of Table 5. For both physical activity and sleep quality evaluations, we formulated the same questions in the present tense when referring to the participants at 3 years of age and in the past tense when referring to them at 1 year of age. In total, four questions were administered for physical activity, and 10 questions were used to assess sleep quality. Table 5 shows the response tendencies for each item of the questions. There were no significant differences between boys and girls in each response. To confirm the reproducibility of the answers, 100 randomly selected participants were asked to answer the same questionnaire 1 week later, and the intraclass correlation coefficient (ICC) for each question item was calculated. The ICC was 0.75 ± 0.09 for questions at the age of 1 year and 0.73 ± 0.11 for questions at the age of 3 years, both of which indicate moderate to high reproducibility.

**Table 5:** Results for analysis of responses to questions about physical activity and sleep quality.

Q	Sex	Mean ± SD	Sig	d	Percentage of respondents (%)			
					1: Not true	2: Not very true	3: True	4: Very true
1-PA1	Total	3.14 ± 0.63			1.44	9.38	62.85	26.33
	Boys	3.14 ± 0.63	ns	0.008	1.56	9.15	63.28	26.02
	Girls	3.14 ± 0.63			1.33	9.61	62.43	26.64
1-PA2	Total	3.22 ± 0.68			1.21	10.69	53.37	34.74
	Boys	3.22 ± 0.66	ns	0.017	0.95	10.50	53.79	34.76
	Girls	3.21 ± 0.69			1.46	10.87	52.95	34.72
1-SQ1	Total	2.46 ± 0.90			16.98	31.59	39.93	11.49
	Boys	2.48 ± 0.89	ns	0.051	16.06	30.76	41.94	11.25
	Girls	2.44 ± 0.92			17.89	32.41	37.97	11.73
1-SQ2	Total	2.37 ± 0.94			20.40	34.37	33.17	12.06
	Boys	2.39 ± 0.94	ns	0.046	19.44	34.42	33.67	12.47
	Girls	2.35 ± 0.94			21.34	34.33	32.67	11.66
1-SQ3	Total	2.54 ± 0.89			15.04	28.31	44.15	12.50
	Boys	2.54 ± 0.88	ns	0.011	14.63	29.00	44.51	11.86
	Girls	2.55 ± 0.91			15.44	27.63	43.80	13.12
1-SQ4	Total	2.31 ± 0.89			20.94	35.21	36.18	7.67
	Boys	2.31 ± 0.87	ns	0.012	20.19	35.64	36.99	7.18
	Girls	2.30 ± 0.90			21.67	34.79	35.39	8.15
1-SQ5	Total	1.95 ± 0.83			32.53	43.79	19.36	4.32
	Boys	1.95 ± 0.82	ns	0.017	32.38	44.78	18.56	4.27
	Girls	1.96 ± 0.84			32.67	42.81	20.15	4.37
3-PA1	Total	3.26 ± 0.68			1.71	8.64	51.56	38.09
	Boys	3.27 ± 0.68	ns	0.019	1.83	7.93	51.83	38.41
	Girls	3.25 ± 0.69			1.59	9.34	51.29	37.77
3-PA2	Total	3.17 ± 0.74			2.55	12.60	50.22	34.64
	Boys	3.17 ± 0.74	ns	0.002	2.24	14.02	48.37	35.37
	Girls	3.17 ± 0.73			2.85	11.20	52.02	33.93
3-SQ1	Total	2.06 ± 0.87			28.81	41.94	23.28	5.96
	Boys	2.09 ± 0.88	ns	0.048	27.78	42.55	22.90	6.78
	Girls	2.04 ± 0.86			29.82	41.35	23.66	5.17
3-SQ2	Total	1.90 ± 0.83			35.51	43.08	17.32	4.09
	Boys	1.89 ± 0.83	ns	0.024	36.65	41.80	17.55	4.00
	Girls	1.91 ± 0.82			34.39	44.33	17.10	4.17
3-SQ3	Total	2.14 ± 0.88			26.97	37.92	29.38	5.73
	Boys	2.15 ± 0.88	ns	0.022	26.90	37.20	30.01	5.89
	Girls	2.13 ± 0.87			27.04	38.63	28.76	5.57
3-SQ4	Total	1.98 ± 0.84			31.52	43.15	20.94	4.39
	Boys	2.00 ± 0.85	ns	0.035	31.50	41.73	22.22	4.54
	Girls	1.97 ± 0.83			31.54	44.53	19.68	4.24
3-SQ5	Total	1.81 ± 0.78			38.19	46.20	12.23	3.38
	Boys	1.80 ± 0.76	ns	0.015	37.94	47.02	11.99	3.05
	Girls	1.81 ± 0.79			38.44	45.39	12.46	3.71

Significant differences between sexes; SD: standard deviation; sig.: significance; ns: no significance; d: Cohen’s d (effect size); 1-: at the age of 1 year; 3-: at the age of 3 years; PA1: often moved around freely by themselves; PA2: often played with their hands; SQ1: experienced difficulty in putting them to sleep; SQ2: cried at midnight; SQ3: had irregularities in sleeping and wake-up times; SQ4: woke up often in the middle of the night; SQ5: often had a bad mood during the day.

Q.: PA: physical activity, SQ: sleep quality.

### Statistical analysis

To determine whether there were sex-specific differences in the tendencies of responses, an independent t-test was performed, and the p-values (significant probability) and Cohen's d (effect size) were determined. In addition, to verify the causal relationship of physical activity and sleep quality at the age of 1 (past)/3 (current) years with psychosocial health at the age of 3 years (current), we tested the causal structural model hypothesized using structural equation modeling. The model parameters were estimated using maximum likelihood. The validity of the hypothetical model was determined according to the following indicators: GFI, AGFI, CFI, RMSEA, and AIC. In this study, referring to previous studies^45^ and textbooks^46, 47^, the following criteria were set for adopting the model: "GFI of 0.95 or more, AGFI close to 1, CFI of 0.95 or more, RMSEA of 0.05 or less, and the smaller the AIC the better." Regarding SDQ, as mentioned earlier, a lower total difficulty score indicates being psychosocially healthier, and conversely, a higher prosocial behavior score does so. To achieve the same interpretation that the higher score shows the better condition in both, on total difficulties, the value after subtracting the score from the perfect score of 40 points was used. On prosocial behavior, the obtained scores were used as it is. Considering a higher applicability to the question to indicate higher physical activity, the two questions on physical activity in the questionnaire were scored as follows: 1 (not true): 0 points, 2 (not very true): 1 point, 3 (true): 2 points, and 4 (very true): 3 points. For the five questions about sleep quality, those that did not fit the questionnaire were considered to have higher sleep quality, where 1 (not true): 3 points, 2 (not very true): 2 points, 3 (true): 1 point, 4 (very true): 0 points. Mixed-sex models were checked to determine whether they met the conformance criteria, and analyses by sex were performed on the verified models in the same manner. The hypothetical model was verified, and the final model was adopted if it met the aforementioned criteria and all path coefficients were significant. If any of the above criteria were not met or any of the path coefficients were not significant, the model was improved by removing the non-significant paths. In Supplementary Information, the hypothesis model is presented in Supplementary Figure S1 and the analysis results in Supplementary Table S1, which were not adopted in this study due to failure in meeting the above conditions. In the above statistical analysis, the significance levels were set at 5%, 1%, and 0.1%.

IBM SPSS Statistics SPSS 25 and IBM SPSS Amos 26 graphics (IBM Corp., Armonk, NY, USA) were used for all statistical analyses.

### Supplementary Information

Supplementary Information.

## Data Availability

The datasets presented in this article are not readily available due to participant confidentiality concerns. To access the datasets, a reasonable request should be directed to the corresponding author.
