# Supplementary material for: Qualitative study of the association between psychosocial health and physical activity/sleep quality in toddlers
Source: Sci Rep. 2023 Sep 21;13:15704. doi: 10.1038/s41598-023-42172-4 (PMC10514029; doi:10.1038/s41598-023-42172-4)
Supplement: Supplementary file 1 — Supplementary Information. [file 41598_2023_42172_MOESM1_ESM.docx]

**Supplementary Information**

**Qualitative study of the association between psychosocial health and physical activity/sleep quality in toddlers**

Chisa Tsuyuki^1, 2*^, Koya Suzuki^1*^, Kanako Seo^1,2^, Dandan Ke^1, 3^, Kyoko Tsuge^1, 4^, Pengyu Deng^1^, Dajiang Lu^5^ and Hisashi Naito^1^

^1^ Graduate School of Health and Sports Science, Juntendo University, 1-1 Hirakagakuendai, Inzai, Chiba 270-1695, Japan

^2^ Tokyo Research Laboratories, Kao Corporation, 2-1-3 Bunka, Sumida-ku, Tokyo 131-8501, Japan

^3^ School of Public Health, Fudan University, Shanghai 200032, China

^4^ Tochigi Research Laboratories, Kao Corporation, 2606 Akabane, Ichikai-machi, Haga-gun, Tochigi 321-3497, Japan

^5^ Department of Human Sports Science, Shanghai University of Sport, Shanghai 200438, China

**^*^Corresponding authors**

[tsuyuki.chisa@juntendo.ac.jp](mailto:tsuyuki.chisa@juntendo.ac.jp) (CT)

[ko-suzuki@juntendo.ac.jp](mailto:ko-suzuki@juntendo.ac.jp) (KS)

This Supplementary Information includes the hypothesis model (**Fig. S1**) and the analysis results (**Table S1**). They were built based on our hypothesis: (1) physical activity at the ages of 1 and 3 years affects psychosocial health at the age of 3 years, however were not adopted in this study due to failure in meeting the acceptance criteria.

The hypothesis model shown in Figure S1 differs from the model finally adopted to verify hypothesis 1 in that it assumes a pathway from physical activity at the age of 1 year to psychosocial health at the age of 3 years. However, as shown in the results of Table S1's total, goodness of fit measures such as goodness-of-fit index (GFI) and root mean square error of approximation (RMSEA) met the inclusion criteria, although the pathway coefficient from physical activity at the age of 1 year to psychosocial health at the age 3 years was not significant. From this, the path from physical activity at the age of 1 year to psychosocial health at the age of 3 years was removed.


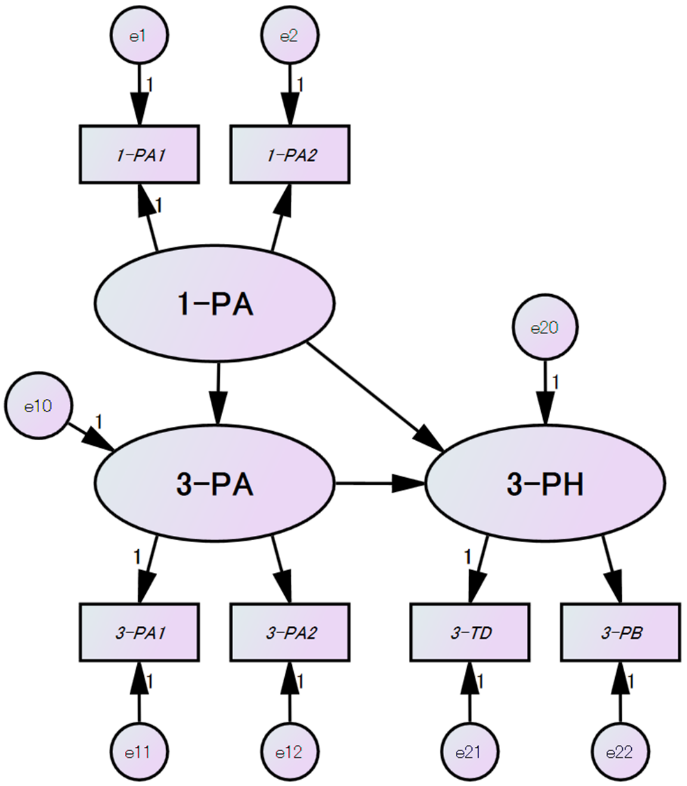


**Supplementary Figure S1** Pathways linking physical activity and psychosocial health

PA: physical activity, PH: psychosocial health, TD: total difficulties, PB: prosocial behavior, e: error term (especially, e10 and e20 in this model mean disturbance terms).

1-: at the age of 1 year; 3-: at the age of 3 years.

PA1: often moved around freely by themselves; PA2: often played with their hands.

**Supplementary Table S1** Goodness-of-fit indices and path coefficients of each structural equation model for physical activity and psychosocial health

|  | Total | | | Boys | | | Girls | | |
| --- | --- | --- | --- | --- | --- | --- | --- | --- | --- |
| GFI | 0.999 | | | 0.998 | | | 1.000 | | |
| AGFI | 0.997 | | | 0.991 | | | 0.999 | | |
| CFI | 0.997 | | | 0.947 | | | 1.000 | | |
| RMSEA | 0.007 | | | 0.023 | | | 0.000 | | |
| AIC | 36.846 | | | 40.841 | | | 31.902 | | |
|  |  |  |  |  |  |  |  |  |  |
|  | *Β* | *β* | Sig. | *Β* | *β* | Sig. | *Β* | *β* | Sig. |
| 1-PA → 3-PA | 0.553 | 0.470 | *** | 0.242 | 0.175 | 0.149 | 0.793 | 0.647 | *** |
| 1-PA → 3-PH | 1.237 | 0.191 | 0.090 | 2.859 | 0.312 | * | 0.489 | 0.072 | 0.716 |
| 3-PA → 3-PH | 2.355 | 0.428 | ** | 2.502 | 0.378 | * | 2.918 | 0.530 | * |
| 1-PA → 1-PA1 | 1.000 | 0.383 | － | 1.000 | 0.267 | － | 1.000 | 0.408 | － |
| 1-PA → 1-PA2 | 0.996 | 0.355 | *** | 1.914 | 0.484 | 0.074 | 0.933 | 0.349 | *** |
| 3-PA → 3-PA1 | 1.000 | 0.414 | － | 1.000 | 0.341 | － | 1.000 | 0.458 | － |
| 3-PA → 3-PA2 | 0.866 | 0.332 | *** | 1.116 | 0.349 | * | 0.792 | 0.340 | *** |
| 3-PH → 3-TD | 1.000 | 0.381 | － | 1.000 | 0.368 | － | 1.000 | 0.432 | － |
| 3-PH → 3-PB | 0.425 | 0.393 | *** | 0.356 | 0.330 | ** | 0.406 | 0.411 | *** |

Path coefficients: partial regression coefficient (*B*), standardized partial regression coefficient (*β*).

Parameter estimation: maximum likelihood.

***: *p* < 0.001, **: *p* < 0.01, *: *p* < 0.05.

GFI: goodness-of-fit index, AGFI: adjusted goodness-of-fit index, CFI: comparative fit index, RMSEA: root mean square error of approximation, AIC: Akaike information criterion, PA: physical activity, PH: psychosocial health, TD: total difficulties, PB: prosocial behavior.

1-: at the age of 1 year, 3-: at the age of 3 years, PA1: often moved around freely by themselves, PA2: often played with their hands.
